# Aging of Low and High Level Vision: From Chromatic and Achromatic Contrast Sensitivity to Local and 3D Object Motion Perception

**DOI:** 10.1371/journal.pone.0055348

**Published:** 2013-01-31

**Authors:** Catarina Mateus, Raquel Lemos, Maria Fátima Silva, Aldina Reis, Pedro Fonseca, Bárbara Oliveiros, Miguel Castelo-Branco

**Affiliations:** 1 Visual Neuroscience Laboratory, IBILI, Faculty of Medicine, Coimbra, Portugal; 2 Department of Ophthalmology, University Hospital of Coimbra, Coimbra, Portugal; University of Montreal, Canada

## Abstract

The influence of normal aging in early, intermediate and high-level visual processing is still poorly understood. We have addressed this important issue in a large cohort of 653 subjects divided into five distinct age groups, [20;30[, [30;40[, [40;50[, [50;60[and [60;[. We applied a broad range of psychophysical tests, testing distinct levels of the visual hierarchy, from local processing to global integration, using simple gratings (spatial contrast sensitivity -CS- using high temporal/low spatial frequency or intermediate spatial frequency static gratings), color CS using Landolt patches, moving dot stimuli (Local Speed Discrimination) and dot patterns defining 3D objects (3D Structure from Motion, 3D SFM). Aging data were fitted with linear or quadratic regression models, using the adjusted coefficient of determination (R^2^_a_) to quantify the effect of aging. A significant effect of age was found on all visual channels tested, except for the red-green chromatic channel. The high temporal low spatial frequency contrast sensitivity channel showed a mean sensitivity loss of 0.75 dB per decade (R^2^_a_ = 0.17, p<0.001), while the lower intermediate spatial frequency channel showed a more pronounced decrease, around 2.35 dB (R^2^_a_ = 0.55, p<0.001). Concerning low-level motion perception, speed discrimination decreased 2.71°/s (R^2^_a_ = 0.18, p<0.001) and 3.15°/s (R^2^_a_ = 0.13, p<0.001) only for short presentations for horizontal and oblique meridians, respectively. The 3D SFM task, requiring high-level integration across dorsal and ventral streams, showed the strongest (quadratic) decrease of motion coherence perception with age, especially when the task was temporally constrained (R^2^_a_ = 0.54, p<0.001). These findings show that visual channels are influenced by aging into different extent, with time presenting a critical role, and high-level dorso-ventral dominance of deterioration, which accelerates with aging, in contrast to the other channels that show a linear pattern of deterioration.

## Introduction

Gradual decline of visual function is a feature of normal aging. This partly results from the combination of optical factors, retinal neural factors such as photoreceptor and ganglion cell degeneration and cortical factors [1], [2]. Many visual functions are expected to be impaired in people in the last aging decades, which is a rising problem due to changes in demographic structure of western populations. Our recent functional magnetic resonance imaging (fMRI) study [3] showed that this process is distinct in healthy and pathological aging.

Few studies are available to study the age dependence of low- and high-level visual function, from local contrast sensitivity (CS) to the ability to integrate local motion cues into object percepts (for a review see Owsley, 2011 [2]). To our knowledge, there are no comprehensive hierarchical studies addressing visual performance from single grating or dot perception to 3D object recognition [4]. Existing studies had a limited scope either with a focus on low-level vision and/or focusing on limited age cohorts [5]–[26]. It is important to note that high-level SFM processing, requiring global integration of coherent motion cues amongst noise, has never been investigated before in normal aging and with such spanning of age groups [4]. In sum, a hierarchical approach of low- and high-level visual function analysis has not been used before to access aging across life span. Here we attempted to address this issue using a hierarchical approach from the point of view of visual processing and stimulus construction. In this way we used chromatic and achromatic contrast sensitivity tasks with simple gratings and patches, motion discrimination tasks with simple random dots kinetograms (RDKs), and high-level object integration tasks using the same type of dots. The rationale of this study tackles the principles of the organization of the visual system. In the early part of the visual system, information is transmitted from the retina to the visual cortex through three parallel and physiologically distinct pathways. The Parvocellular (P) pathway responds to achromatic static stimulation of relatively high spatial frequency and also underlies for color perception in the red-green axis. The Koniocellular (K) pathway conveys information concerning blue-yellow opponency [27], [28]. The Magnocellular (M) pathway is most sensitive to achromatic stimuli with low spatial and high temporal frequencies and underlies local motion processing [6], [21]. This pathway represents the main projection to the visual dorsal stream (parietal), where motion integration takes place [3].

To understand how aging affects the visual function across multiple organization levels we have used low-level stimulation paradigms that preferentially activate the red-green (P) and blue-yellow (K) channels (chromatic CS approach, Cambridge Color Test, CCT [28], [29], [30]), an achromatic channel tuned to Intermediate Spatial Frequencies (ISF [31], [32], [33]); a achromatic channel biased for the M pathway (Frequency Doubling Technology testing, FDT [32]); and a local motion perception (M-biased) channel (Local Speed Discrimination Test, LocSp [34], [35]). Finally we addressed high-level motion integration and object perception using a 3D task (Structure from Motion, SFM) requiring high-level motion coherence [34], [35], [36], [37]. This task targets dorsal stream function and dorso-ventral integration for object recognition [3], [36].

## Methods

### Ethics Statement

This study and all procedures were reviewed and approved by the Ethics Commissions of the Faculty of Medicine of the University of Coimbra (“Comissão de Ética da Faculdade de Medicina da Universidade de Coimbra”) and were conducted in accordance with the Declaration of Helsinki. Written informed consent was obtained from each participant and procedures of the study were fully explained.

### Participants

To analyze the effect of age in low- and high-level visual performance we have used five psychophysical tests: ISF and FDT perimetry, CCT, LocSp and 3D SFM. All tests were performed in a darkened room.

Demographic data for each psychophysical test was as follows: FDT, 158 subjects (mean age: 41.68±1.26 [SEM] years, range: 20–83); LocSp, 97 subjects (mean age: 45.36±1.62 years, range: 20–77); SFM, 98 subjects (mean age: 45.39±1.60 years, range: 20–77); CCT, 135 subjects (mean age: 39.65±1.35 years, range: 20–71); and ISF, 165 subjects (mean age: 39.81±1.21 years, range: 20–72). Only the dominant eye of each subject was tested, except for SFM that was performed binocularly. These data were divided into five age groups: [20;30[, [30;40[, [40;50[, [50;60[and [60;[. There were no statistical differences between gender, eye and education level of participants (demographic characteristics are summarized in Table 1).

**Table 1:** Demographic characteristics of the population study.

Test	Age groups	Gender (male:female)	N (subjects)	Mean age/SEM (years)	Range
**FDT**	[20;30[	14∶29	43	24.84/0.43	20–29
	[30;40[	20∶24	44	33.64/0.41	30–38
	[40;50[	7∶13	20	44.30/0.62	40–48
	[50;60[	6∶14	20	54.90/0.69	50–59
	[60;[	16∶15	31	66.23/1.07	60–83
**LocSp**	[20;30[	8∶12	20	24.50/0.56	20–29
	[30;40[	6∶13	19	34.00/0.66	30–39
	[40;50[	10∶9	19	45.32/0.56	41–49
	[50;60[	8∶11	19	54.37/0.67	50–59
	[60;[	6∶14	20	68.50/1.01	63–77
**3D SFM**	[20;30[	8∶12	20	24.50/0.56	20–29
	[30;40[	6∶13	19	34.00/0.66	30–39
	[40;50[	10∶10	20	45.45/0.55	41–49
	[50;60[	8∶11	19	54.37/0.67	50–59
	[60;[	6∶14	20	68.50/1.01	63–77
**CCT**	[20;30[	16∶34	50	23.56/0.34	20–29
	[30;40[	4∶19	23	32.91/0.56	30–38
	[40;50[	4∶16	20	46.20/0.63	41–49
	[50;60[	8∶17	25	55.60/0.59	51–59
	[60;[	7∶10	17	64.94/1.01	60–71
**ISF**	[20;30[	20∶42	62	24.45/0.32	20–29
	[30;40[	8∶22	30	33.00/0.46	30–39
	[40;50[	13∶10	23	46.30/0.53	41–49
	[50;60[	6∶18	24	54.67/0.65	50–59
	[60;[	9∶17	26	64.81/0.61	61–72

Note. FDT = Frequency Doubling Technology; LocSp = Local Speed Discrimination; 3D SFM = 3D Structure from Motion; CCT = Cambridge Color Test; ISF = Intermediate Spatial Frequency Perimetry; SEM = standard error of the mean.

For each participant, only the dominant eye was performed, except for SFM that was tested binocularly.

Participants were submitted to a complete ophthalmic examination, including best corrected visual acuity (VA) obtained with Snellen chart (observers were refracted for the target distance of each test), ocular tension (Goldman applanation tonometer), slit lamp biomicroscopy and fundus examination (Goldman lens).

Exclusion criteria included retinal and neurological diseases, optic nerve pathology, diabetes even in the absence of retinopathy, significant media opacities that precluded fundus examination (subjects with incipient cataract were included), high ammetropy (sphere>±4D; cylinder>±2D) and congenital color vision defects (to screen for congenital red-green deficiency the Ishihara Pseudoisochromatic Plates was employed). Older adults (>60 years) were tested with the Mini-Mental-State examination, in order to exclude for dementia. No subject showed any signs of Age-Related Macular Degeneration (even in an early stage).

### Intermediate Spatial Frequency Perimetry – Low-level Achromatic Contrast Sensitivity

We have applied CS multiple interleaved staircase testing with ISF perimetry [32], [33] at multiple locations (9 locations: 1 central, 4 paracentral and 4 peripheral), where stimuli were patches of 3.5 cpd (cycle per degree) of vertically oriented sinusoidal gratings and 0 Hz temporal frequency, displayed on a gamma corrected 21 inch Trinitron GDM-F520 Sony color monitor (frame rate 100Hz) with background luminance of 51 cd/m^2^ at a viewing distance of 36 cm. Implementation and calibration procedures were performed with software and hardware provided by CRS (Minolta colorimeter; calibration software and CRS/VSG 2/5 graphics card, with 15-bit contrast resolution per pixel). An adaptive logarithmic staircase strategy was used to obtain psychophysical thresholds. Staircases were run for a total of four reversals, with the contrast at the final two reversals being averaged to estimate the contrast threshold. Results were expressed in terms of decibels (dB) units.

This spatial testing was performed monocularly (dominant eye was tested). Subjects were instructed to fixate a black square (1°×1°) in the centre of the screen and report the presence of vertical “striped” targets by means of a button press. Stimulus duration was 200 ms and interstimulus interval was jittered between 2300–2800 s. Participants reliability was evaluated by the inclusion of false positive and negative “catch trials”, and all results with false positive and false negative errors ≥33% were excluded. Fixation loss was monitored with our custom eye-tracking methodology (CRS device) which provides detailed measurements of eye position.

For analysis, 4 visual field quadrants (IN, inferior nasal; IT, inferior temporal; SN, superior nasal; ST, superior temporal) and 3 zones were defined: zone 1 corresponds to 5° of visual field, zone 2 contains locations between 5° and 10° eccentricity and zone 3 contains locations between 10° and 20° eccentricity.

### Frequency-Doubling Technology Perimetry – Testing a Low-level Achromatic High Temporal and Low Spatial Frequency Channel

FDT perimeter (Humphrey Matrix perimeter, Welch Allyn, Skaneateles, NY; Zeiss – Humphrey, Dublin, CA) determines CS measures for detection of sinusoidal gratings with spatial frequency of 0.25 cpd undergoing counterphase flicker at 25Hz [32], [38]. We performed N-30-F FDT full threshold test, which uses a staircase threshold strategy known as a Modified Binary Search (MOBS) with a four-reversals rule for determining the threshold level. The range of possible threshold level values for the raw data is between 0 dB Maximum Contrast and 56 dB Minimum Contrast. Stimulus duration was 300 ms and background luminance was 100 cd/m^2^. Stimuli (10° by 10° squares, except for a 5° radius central circular target) were presented at 17 locations, plus 2 nasal locations, testing 30° nasally and 20° temporally. Performance reliability was assessed by monitoring fixation loss and computing false positive and negative errors (results with false positive and negative errors ≥33% and fixation loss ≥20% were excluded [39], [40]). In this study, subjects were instructed to fixate the black square in the centre of the screen and report the presence of “striped” targets. All participants performed the test under monocular conditions.

For analysis purposes, 4 visual field quadrants (IN, IT, SN, ST) and 3 zones were defined: zone 1 corresponds to 5° of visual field, zone 2 contains locations between 5° and 10° eccentricity and zone 3 contains locations between 10° and 20° temporally or 30° nasally.

### Cambridge Color Test - Red-green (P) and Blue-yellow (K) Channels

We have probed P and K pathways using a slightly modified version of CCT (Cambridge Research Systems Lda., CRS, Rochester, UK) [28], [29], [30]. This technique measures chromatic CS using three parallel, randomly interleaved staircases, corresponding to simultaneous assessment of the three cone confusion axes (protan, deutan and tritan) modulated in the CIE 1976 u‘v’ color space (Trivector version of the test). Each staircase was composed by 11 reversals and the mean of the last 7 reversals was taken as the threshold estimate. Subjects looked monocularly at a screen (viewing distance –180 cm) with a pattern of disks of various sizes and luminance with superimposed chromatic contrast defining a Landolt-like C-shaped ring. Luminance and size variation of stimulus patches forced the subject to use specific color cues, since he/she could not use spatial or luminance cues to infer the embedded shape. These patches were randomly assigned six different luminance noise levels (8 to 18 cd/m^2^ in steps of two units). Subjects were instructed to indicate one out of four possible gap positions (bottom, top, left and right) of the Landolt C stimulus, and the experimenter recorded subjects’ oral responses using a 4-button response box. Psychophysical thresholds were expressed in CIE 1976 u‘v’ color space units.

### Local Speed Discrimination Test - Low-level Local Motion Perception

Motion stimuli (random single dots) were generated using Vision-Works™ for Windows (Vision Research Graphics, Wisconsin, USA) in a Trinitron GDM-F520 monitor with refresh rate set to 75 Hz. Viewing distance was 56 cm. Pixel size was 0.056 degree^2^ and dot size was 3*2 pixels (∼0.07°). The background luminance was ∼0 cd/m^2^ and each pixel had approximately 18 cd/m^2^ (these specifications also hold for the 3D Structure from Motion test, see below).

A computerized LocSp test was developed to measure local motion sensitivity [34], [35]. It is based on velocity comparisons of two widely separated dots moving with random trajectories within a circular spatial window (2° of diameter aperture), across two meridians (horizontal (HM) 0° and oblique meridian (OM) 45°). At HM a relatively small eccentricity was used (7.5°), while dot eccentricity in the OM was 15°. Each test consisted of two apertures on the same meridian (with a single dot moving randomly inside of each of them) and equal eccentricity with the fixation point located halfway between them. The average luminance of each aperture was 0.13 cd/m^2^, while each single dot had a brightness value of 15% of the maximum output of each of the red, green and blue CRT guns. Fixation point was a white (0.34 cd/m^2^) 0.4° cross-hair with 0.05° arm thickness. The initial speed of the test stimulus was 50°/s and the standard stimulus was moving at 15°/s. Two time conditions were used (400 and 600 ms). Subjects responded verbally (left/right, to prevent issues related to aging) which was the fastest moving dot and the experimenter introduced the response. Subjects were instructed to keep their eye fixed (exam tested under monocular conditions) on the fixation point at the centre of screen. A two-alternative forced-choice staircase method was used, with a total of 12 reversals, 6 practice and 6 experimental (which were used to calculate the average threshold). The results were given as a geometric mean of the experimental reversals in threshold units (°/s).

### 3D Structure from Motion – High-level Motion Coherence Performance

A very simple task was used, where subjects just needed to report the presence of a “ball or sphere”. This task was very easy to perform because the “sphere” becomes very noticeable, with a strong 3D percept, once stimuli are above threshold [36]. Comprehension and performance can be controlled by analyzing the shape of the curve of the psychophysical staircase (if the subjects understand the task, the staircase converges in a monotonic way to asymptotic threshold levels). This represents a methodological asset because one can objectively control for correct task performance. We also had a familiarization session prior to the formal test and made sure that all subjects understood the task. This was then verified by analysis of the psychophysical staircase.

Duration of fixed stimuli presentation was 200 ms (fixed interval, FI) in the main test and a control test allowed for stimuli present until response (UR), after which a gray background appeared.

Visual thresholds units were measured in terms of % motion coherence needed to detect spherical surfaces. The stimulus consisted of dots placed on the surface of a rotating sphere 3D in diameter revolving around an imaginary axis, which angle varied in a pseudo-random way. Speed of revolution was 20 rpm. The sphere alternated within one aperture with a stimulus consisting of 100% noise dots that moved at 2°/s. Subjects had to report the presence or absence of a rotating sphere (under binocular conditions) and responses were recorded by the experimenter, using a button response box.

### Statistical Analysis

Statistical analysis was performed with the SPSS statistical software package, version 19.0 (SPSS, Inc., Chicago, IL). When data significantly deviated from normal distributions (verified using the Kolmogorov-Smirnov normality check and Levene homogeneity tests), we applied non-parametric statistical methods. Results with *p*<0.05 were considered statistically significant. Comparisons between means were performed with the use of the general linear model [univariate analysis of variance (ANOVA)] with Bonferroni’s adjustment for multiple comparisons where appropriate or the Kruskal-Wallis 1-way ANOVA for k samples with pairwise comparisons. When applicable, correlation analysis (Spearman or Pearson coefficients) were performed between each functional quantitative parameter and age. Low-level visual performance as a function of age (by age group) was fit with linear regression and high-level dorsal stream performance was fit with a quadratic regression model. We used as an indicator of goodness of fit and measure of age effect the adjusted coefficient of determination (R^2^_a_).

## Results

### Functional Evaluation of Low-level Visual Age-related Changes

#### ISF perimetry (Intermediate spatial frequency channel)

**Figure 1:**
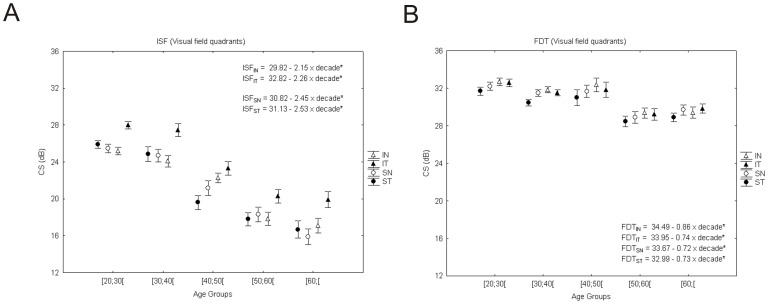
Achromatic contrast sensitivity performance over normal aging (analysis by quadrants). A decrease in contrast sensitivity using ISF perimetry (A) was more evident than that found using M-biased perimetry (B). Error bars represent standard error of the mean (SEM). The dependent measure corresponds to CS measured in decibel units (* corresponds to p<0.001, significant regression equations depicted here and in subsequent figures with regression data). IN - Inferior nasal; SN - Superior nasal; IT - Inferior temporal; ST - Superior temporal.

**Figure 2:**
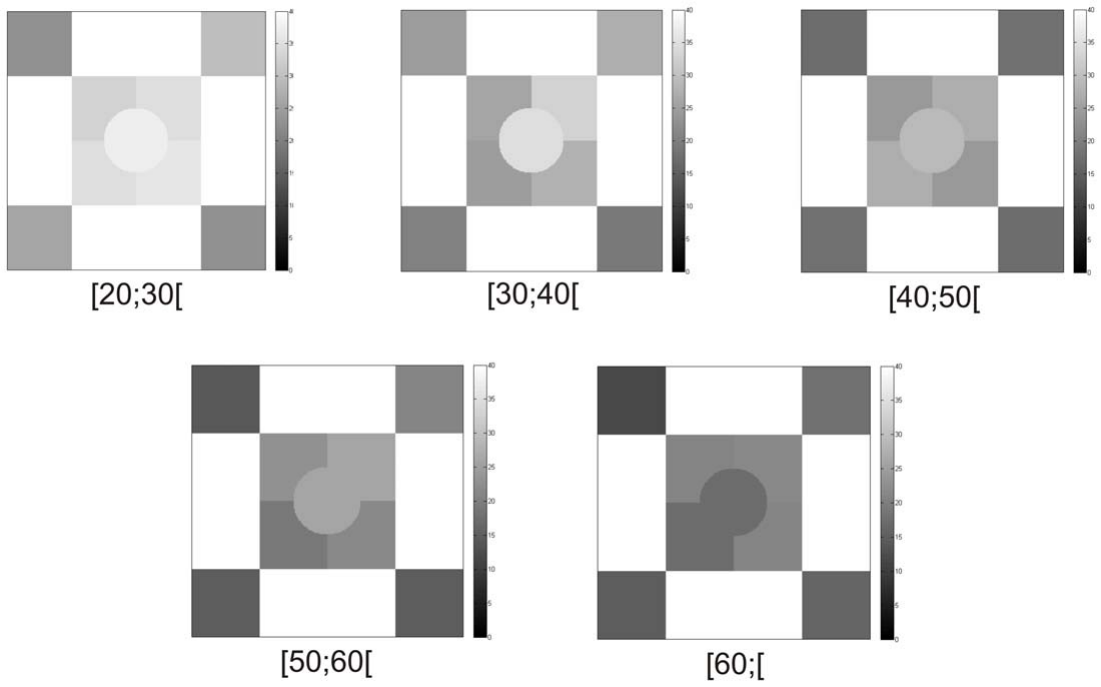
Illustration of progressive sensitivity loss in achromatic CS over age groups assessed by ISF perimetry, using representative cases for each age group. Grayscale bar depicts contrast sensitivity in decibels. Darker regions correspond to worse CS.

We measured achromatic CS at an intermediate spatial frequency for nine visual field locations (see Figure 1A for data across quadrants and Figure 2 for representative examples of age-related CS decline obtained with ISF perimetry for each age group) and found a highly significant effect of aging on mean CS [F_(4,164)_ = 53.09, p<0.001]. Post-hoc tests revealed significant differences between all groups (p<0.02), except for [50;60[v.s. [60;[groups. Furthermore, as expected, a strong monotonic correlation between age and mean CS was present (rho = -0.72, p<0.001), whereby CS performance declines with aging. Then, we applied a linear regression to data and found that mean CS decreases linearly 2.35 dB per age group (y = 31.81–2.35*age group, F(1,163) = 202.81, R^2^_a_ = 0.55, p<0.001) and 55% of the variability in CS can be explained by aging.

We also studied the effect of aging in this P-biased CS test in terms of eccentricity: CS declined more for the two central tested zones (Zone1: −2.39 dB, R^2^_a_ = 0.27, p<0.001; Zone2: −2.68 dB, R^2^_a_ = 0.49, p<0.001), and was less pronounced in the most peripheral zone (Zone3: −2.02 dB, R^2^_a_ = 0.51, p<0.001). Along visual field quadrants, CS decreased between 2.15 dB and 2.53 dB per age group (SN: 2.45 dB, R^2^_a_ = 0.47; IN: 2.15 dB, R^2^_a_ = 0.47; ST: 2.53 dB, R^2^_a_ = 0.49; IT: 2.26 dB, R^2^_a_ = 0.44; p<0.001 for all cases; see Figure 1A).

#### FDT perimetry

**Figure 3:**
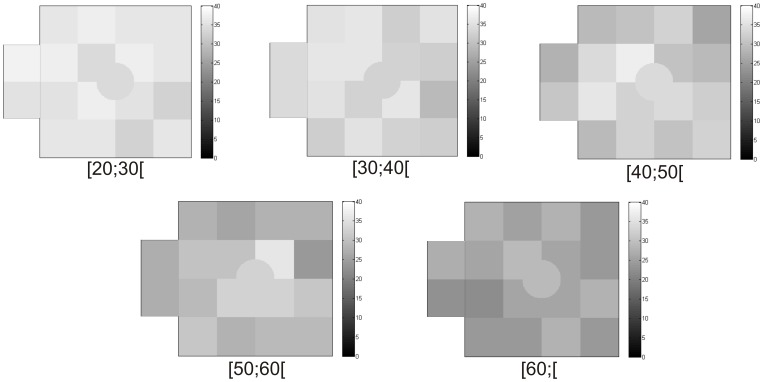
Illustration of gradual decline in achromatic CS over age groups observed with FDT perimetry using representative cases for each age group. Grayscale bar depicts contrast sensitivity in decibels. Darker regions correspond to worse CS.

We found also a significant effect of aging in M-biased CS (dB) [F_(4,152)_ = 10.3, p<0.001] (see Figure 1B for visual quadrant comparisons and Figure 3 for representative examples). In fact post-hoc tests showed significant differences between the three younger age groups and the two older ones ([20,30[, [30;40[, [40;50[and [50;60[, [60;[(p<0.02). This low spatial/high temporal frequency channel showed only a moderate correlation with age (CS decreases with aging, rho = −0.49, p<0.001). Mean CS loss was approximately linear with 0.75 dB per age group (y = 33.83–0.75*age group, F_(1,155)_ = 32.8, with R^2^_a_ = 0.17, p<0.001) with only 17% of variability in CS explained by age (see Figure 3 for representative figure of gradual decline in CS across age groups). We also studied the effect of aging in terms of eccentricity for M-biased CS and its loss was around 0.49 dB per age group for the most central location (Zone1: R^2^_a_ = 0.04, p = 0.005) and increased to 0.82 dB for the more peripheral locations (Zone3: R^2^_a_ = 0.19, p<0.001), while for intermediate locations it was 0.54 dB (Zone2: R^2^_a_ = 0.07, p<0.001). Moreover, data in terms of visual field quadrants was fitted with linear regressions (Figure 1B). The IN (inferior nasal) quadrant showed the most pronounced decrease of CS per age group (IN: 0.86 dB, R^2^_a_ = 0.18; SN (superior nasal): 0.72 dB, R^2^_a_ = 0.13; ST (superior temporal): 0.73 dB, R^2^_a_ = 0.13; IT (inferior temporal): 0.74 dB, R^2^_a_ = 0.14; p<0.001 for all cases).

#### Color vision

**Figure 4:**
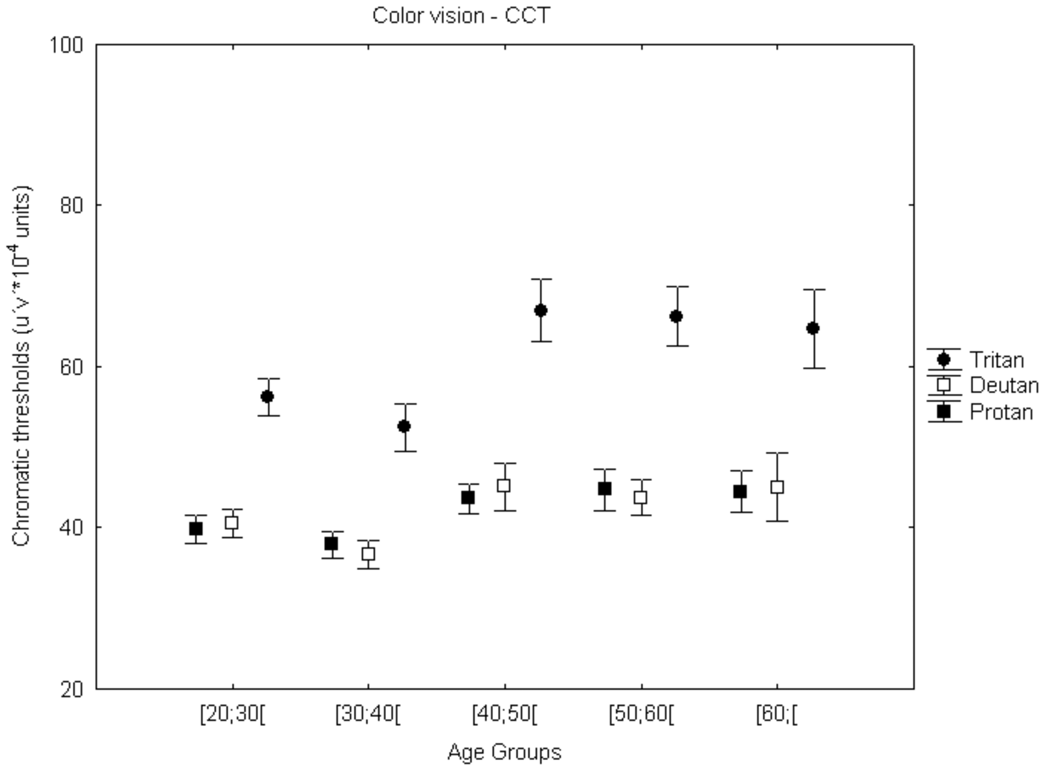
Color vision (red-green and blue-yellow channels) across normal aging. A trend for loss in chromatic performance was identified from [30;40[to [40;50[(marginally significant for tritan axes). Error bars depict standard error of the mean (SEM). The dependent measure is color excursion units measured in CIE u‘v’ color space units (note that high chromatic thresholds relate to low CS).

Blue-yellow (K) sensitivity deteriorates more, as expected, than red-green (P) contrast sensitivity for all age groups and this change was more pronounced between [30;40[and [40;50[(see Figure 4, note that high chromatic thresholds relate to low CS). We performed the Kruskal-Wallis test to assess the age effect across all five age groups. The age effect for protan and deutan thresholds across groups was not significant. However, tritan thresholds did reveal a significant age effect [χ^2^_(4)tritan_ = 12.70, p = 0.013], though post hoc (Dunn-Bonferroni) tests revealed only a marginally significant difference between two age groups ([30;40[vs [40;50[; p = 0.074). Figure 4, does indeed suggests a deterioration of performance around age 40 which was more pronounced for the tritan axes.

### Local Speed Discrimination Test

**Figure 5:**
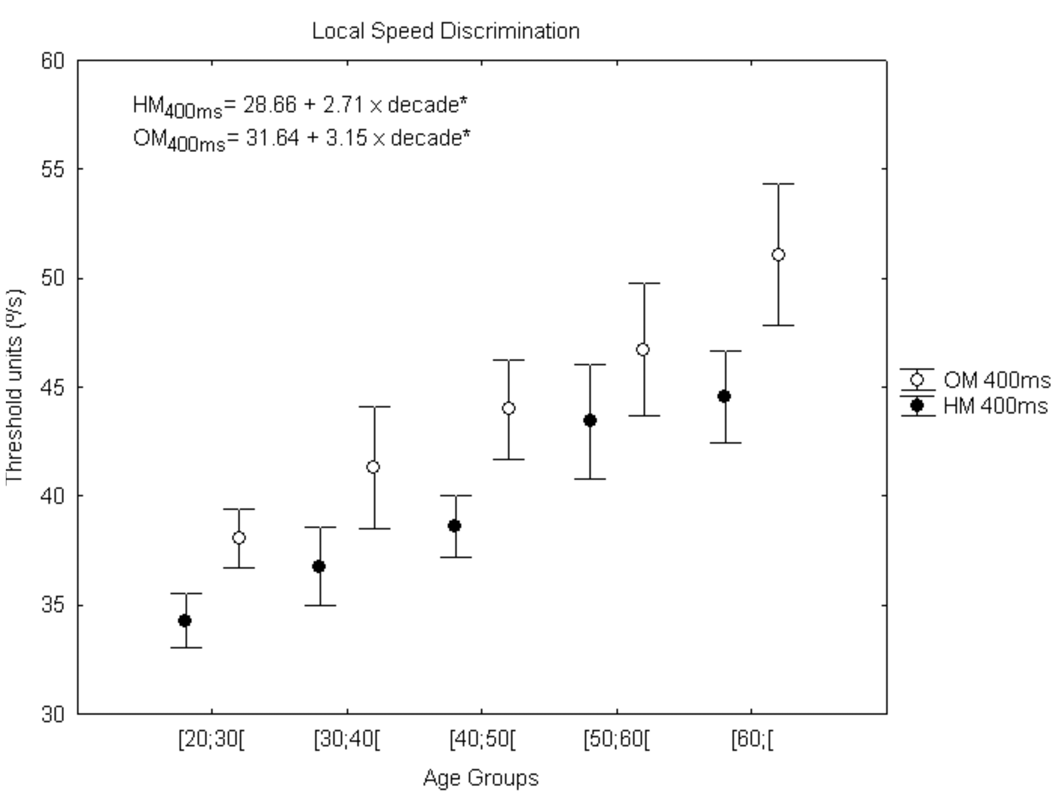
Age-related changes in low-level motion perception using the Local Speed test. Error bars depict standard error of the mean (SEM). The dependent measure corresponds to geometric mean of the experimental reversals in threshold units (°/s) (*p<0.001). OM, oblique meridian; HM, horizontal meridian.

Aging was also studied with a low-level M task in terms of motion perception, using the local speed discrimination (LocSp) test. Interestingly, we found that aging affects speed discrimination for both meridians, but only for the shortest stimulus presentation of 400 ms and not for the longer presentation time of 600 ms [HM_400ms_: F_(4,97)_ = 5.40 (p = 0.001); OM _400ms_: F_(4,97)_ = 3.69 (p = 0.008)]. *Post-hoc* tests showed significant differences between the cohorts of age groups for the following meridians: HM_400ms_ − [50;60[vs.[20;30[(p = 0.009), [60;[vs.[20;30[(p = 0.002); [60;[vs.[30;40[(p = 0.045) and OM _400ms_ − [60;[vs. [20;30[(p = 0.006). We also performed a correlation analysis, to better capture the pattern of decline with age and moderate but very significant correlations were found for both meridians (HM 400 ms: rho = 0.41, p<0.001; OM 400 ms: rho = 0.34, p = 0.001). Thus, threshold values increase linearly (corresponding to a performance decline) with age, around 2.71°/s per age group for HM_400ms_ [F_(1,95)_ = 21.45, R^2^_a_ = 0.18, p<0.001] and 3.15°/s for OM_400ms_ [F_(1,95)_ = 15.09, R^2^_a_ = 0.13, p<0.001], see linear equations in Figure 5. In fact, 18% and 13% of variability in motion perception is explained by age, for HM and OM respectively.

### Functional Assessment of High-level Dorsal Stream Performance

#### 3D Structure from motion coherence

**Figure 6:**
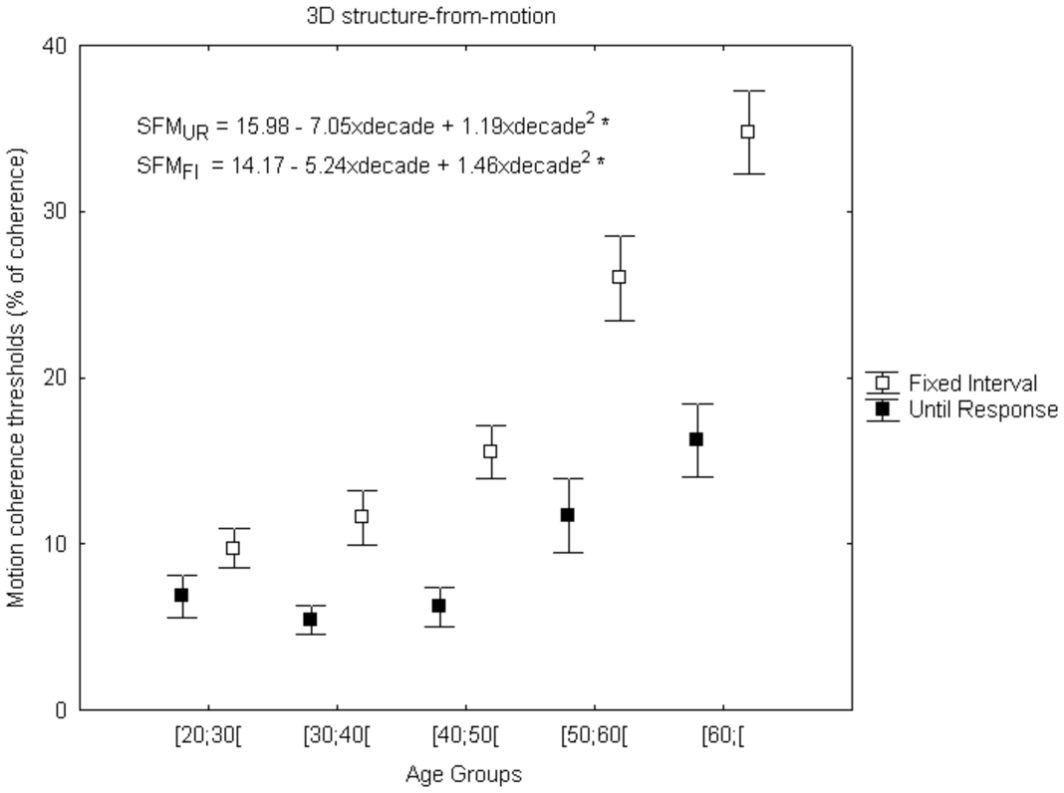
Visual dorsal stream performance during normal aging. High-level functions showed a fast deterioration with an accelerating pattern. Error bars depict standard error of the mean (SEM). The dependent measure is % of coherence (percentage of signal dots that are necessary for the subject to report the presence of a sphere) (*p<0.001). UR, until response; FI, Fixed Presentation Interval.

In order to investigate whether aging could influence high-level dorsal stream performance, we used a novel 3D visual task that probes the ability to perceive 3D spheres by integrating motion signals (cues) immersed in noise motion signals. Nonparametric analyses (Kruskal-Wallis test) were carried out to analyze the age effect across all five groups under two temporal conditions [SFM_FI_: χ^2^_(4)_ = 51.22(p<0.001) and SFM_UR_: χ^2^_(4)_ = 23.19 (p<0.001)]. Thereafter Dunn-Bonferroni corrections for multiple comparisons were performed between the cohorts of age groups and the following significant differences were identified: SFM_FI_ – [20;30[vs.[50;60[, [20;30[vs.[60;[, [30;40[vs.[60;[(p<0.001); [30;40[vs.[50;60[(p = 0.003) and for SFM_UR_ – [20;30[vs.[60;[(p = 0.006); [30;40[vs.[60;[, [40;50[vs.[60;[(p = 0.001). Spearman correlation analysis showed that aging was significantly stronger in the temporally constrained condition of 200 ms [SFM_FI_: rho = 0.71 (p<0.001); SFM_UR_: rho = 0.41 (p<0.001)], which suggests that time is an age related limiting factor for performance in the SFM task. In Figure 6, one can observe a significant difference between the two curves for short and unlimited temporal presentations and that this difference increases with age. In addition, both SFM data suggest an accelerating pattern of deterioration and were fitted with a quadratic regression model (Figure 6). Using the R^2^_a_ parameter, we found that 54% and 23% of variability in 3D SFM coherence perception is explained by age for FI and UR temporal conditions respectively [SFM_FI_: F(2,95) = 58.85, R^2^_a_ = 0.54 (p<0.001) and SFM_UR_: F(2,95) = 15.67, R^2^_a_ = 0.23 (p<0.001)].

## Discussion

In this study, we performed for the first time a hierarchical analysis of low- and high-level visual function within multiple visual channels in normal aging, from early adulthood up to old age cohorts.

It is well known that 3D tasks (requiring the detection of complex SFM targets) involve high-level dorsal stream function and dorso-ventral integration for object recognition [3], [36]. On the other hand, local speed signals are mainly processed in early human visual cortex, in particular V1 and V2 [41]. Finally, chromatic and achromatic contrast sensitivity tests require the integrity of low-level retinal or retinocortical visual pathways [28], [31], [42]. Direct comparisons of performance in these tasks that differentially require low- and high-level visual processing can therefore help inferring on how distinct neural pathways are affected by the aging process. Our major finding was that high-level functions, such as 3D object integration, deteriorate faster with aging, with a quadratic pattern, instead of the linear one observed for other tasks.

It should be taken into account that both optical factors and retinal/cortical neural factors are both characteristics of normal aging. In particular, at photopic light levels, spatial CS deficits may also be affected by optical characteristics [2]. Aging of the lens is particularly relevant in which concerns color vision. Lens yellowing might indeed affect the interpretation of changes in one of our particular tests (blue-yellow discrimination measures) [28], [30]. However, direct comparison with tests in which yellowing of lens does not play a role (along deutan and protan axes), allowed to disentangle these effects. In our study, red-green (P) and blue-yellow (K) contrast sensitivity testing showed only significant age effects in the latter. This seems to be consistent with the relatively stable anatomical cone distribution during aging [43] and also with evidence that optical factors (yellowing of the crystalline lens) contribute for the gradual decline of color vision function with aging, only for stimuli modulated along the tritan axis [44].

To our knowledge no study before used this comprehensive array of testing at single or multiple age groups. In our study, M-biased perimetry (tuned to high temporal and low spatial frequencies) showed that mean sensitivity loss was approximately linear (0.75 dB per age group) and was about three-fold inferior to that found in ISF perimetry (which has a slight peripheral P bias [32]). Interestingly, M testing showed comparatively larger aging deficits for greater eccentricities, unlike ISF where loss dominated in the center. A few previous studies that used particular techniques such as FDT (magnocellular) perimetry to measure achromatic contrast sensitivity suggested that loss was approximately linear at 0.6 dB [7] or similarly, about 0.7 dB per age group [8]. This average rate of decline is consistent with our own data. Concerning the other tested spatial frequencies (ISF test, using higher spatial frequencies), which showed the above reported much faster rate of decline, no similar studies are available. In fact, some aging studies reported a decrease in CS under photopic conditions [10]–[15], but never to our knowledge studied along different visual field locations.

Age-related changes in chromatic CS have been scarcely documented using CCT and only for limited age ranges (between 2 and 30 years by Goulart et al. [45]; 20 and 59 years by Paramei [26]). This limits direct comparison, although our results are consistent with the notion of just a relatively small and subtle decline when all axes are considered.

We studied motion perception, because of the known delay in visual processing of speed, which is a characteristic of aging [16]–[25]. In fact, there is a marked effect of aging on visual motion processing, with older adults exhibiting decreased ability in speed discrimination using sinusoidal gratings [19], [23], [24], detection of direction of movement [20], kinetogram speed [22] and perception of motion coherence [17], [18], [21]. There are scarcely any studies using local speed discrimination tests, using single dots, rendering direct comparison with motion integration tasks difficult. Most previous studies used stimuli such as sinusoidal gratings or multiple dot kinetograms. We have previously used our paradigm in previous pathological aging studies [35], [46]. Our low-level motion perception test using single dots showed age-related deficits specifically for the shorter exposure time of 400 ms. In general the pattern of age related decline is not as severe as the observed with structure from motion stimuli.

Concerning perception of complex moving objects (structure from motion), the pattern of decline was much more severe. This was an important finding because this accelerated high-level deterioration has remained hitherto unreported. This is one of the main novelties of the paper, extending our own previous use of this paradigm in normal elderly and pathological aging populations [3], [36].

In sum, the mainly affected level of processing that was tested was high-level visual dorsal-ventral integration using the 3D SFM task, which has never been investigated before in normal aging. The stimulus used in SFM induces the perception of a 3D rotating sphere, in other words, one perceives a form or structure based on motion information, so an interaction between motion processing areas and brain areas involved in form processing is required [4]. Interestingly, these high-level processes, proved to be time constrained, suggesting that older people require longer time to process motion direction and integration, thereby corroborating the processing speed hypothesis [2], [47] and extending our previous results concerning pathological aging [3], [36].

Taken together our findings suggest that aging influences higher-level vision functions into a disproportionate level [48]. The hierarchical age-related decline of visual functions is further substantiated by the fact that high-level mechanisms showed a quadratic profile of loss, unlike low-level mechanisms which in general showed a linear pattern of decay. Only the low-level achromatic intermediate frequency CS channel showed comparatively high loss, but with a still linear pattern.

Finally, we have found that high-level functions, such as 3D object integration, deteriorate with an accelerating pattern with aging and with increasing temporal constraints, surprisingly departing from the linear pattern observed for low-level functions, and suggest that time and integration variables are critical functional aspects in cortical normal aging.
